# Zebrafish Patient-Derived Xenograft Model as a Preclinical Platform for Uveal Melanoma Drug Discovery

**DOI:** 10.3390/ph16040598

**Published:** 2023-04-15

**Authors:** Jie Yin, Gangyin Zhao, Helen Kalirai, Sarah E. Coupland, Aart G. Jochemsen, Gabriel Forn-Cuní, Annemijn P. A. Wierenga, Martine J. Jager, B. Ewa Snaar-Jagalska, Arwin Groenewoud

**Affiliations:** 1 Institute of Biology, Leiden University, 2333 BE Leiden, The Netherlands; j.yin@biology.leidenuniv.nl (J.Y.);; 2 Liverpool Ocular Oncology Research Centre, Department of Molecular and Clinical Cancer Medicine, University of Liverpool, Liverpool L69 3BX, UK; 3 Department of Cell and Chemical Biology, Leiden University Medical Center, 2333 ZA Leiden, The Netherlands; 4 Department of Ophthalmology, Leiden University Medical Center, 2333 ZA Leiden, The Netherlands; 5 Experimental Renal and Cardiovascular Research, Department of Nephropathology, Institute of Pathology, Friedrich-Alexander-Universität Erlangen-Nürnberg (FAU), 91054 Erlangen, Germany

**Keywords:** eye, oncology, uveal melanoma, xenograft, zebrafish, drug toxicity, drug screening

## Abstract

Uveal melanoma (UM) is a rare malignant cancer of the eye, with up to 50% of patients dying from metastasis, for which no effective treatment is available. Due to the rarity of the disease, there is a great need to harness the limited material available from primary tumors and metastases for advanced research and preclinical drug screening. We established a platform to isolate, preserve, and transiently recover viable tissues, followed by the generation of spheroid cultures derived from primary UM. All assessed tumor-derived samples formed spheroids in culture within 24 h and stained positive for melanocyte-specific markers, indicating the retention of their melanocytic origin. These short-lived spheroids were only maintained for the duration of the experiment (7 days) or re-established from frozen tumor tissue acquired from the same patient. Intravenous injection of fluorescently labeled UM cells derived from these spheroids into zebrafish yielded a reproducible metastatic phenotype and recapitulated molecular features of the disseminating UM. This approach allowed for the experimental replications required for reliable drug screening (at least 2 individual biological experiments, with *n* > 20). Drug treatments with navitoclax and everolimus validated the zebrafish patient-derived model as a versatile preclinical tool for screening anti-UM drugs and as a preclinical platform to predict personalized drug responses.

## 1. Introduction

Uveal melanoma (UM) is a rare intraocular disease with an incidence of 5.1 cases per million individuals per year in Europe and the United States. It is the most common ocular malignancy in adults [1]. The uveal tract consists of the iris, ciliary body, and choroid. UMs originate from melanocytes in these tissues, with an incidence of 90% in the choroid, 6% in the ciliary body, and 4% in the iris [2]. Clinically, early stage UM patients, are treated with various forms and combinations of radiotherapy, phototherapy, and local resection. Treatment generally aims to conserve the eye and useful vision, reserving enucleation for advanced cases [3]. Despite the treatment of primary UM, up to 50% of patients will develop metastatic disease, with the vast majority occurring in the liver. The survival of most patients with metastasized UM is less than 5 years [4,5]. Currently, there is a lack of effective therapies to either prevent or treat UM metastasis. Over the past few decades, the prognosis for UM patients has not changed. Generally accepted variables estimating the prognosis for UM include the patient’s age, the size of the tumor, and histology [6,7]. Even with an early diagnosis, appropriate treatment, and close follow-up, an estimated 40–50% of all patients will eventually die of metastatic diseases [8,9,10]. Therefore, it is vital to study the primary UMs, to discover mechanisms of metastasis and to develop new therapeutic strategies.

In recent years, primary UM tissues have gained more attention in UM research, considering that various UM cell lines may not represent the full molecular heterogeneity of primary tumors [11,12]. As part of the UM CURE 2020 project, we proposed that it is necessary to establish preclinical models recapitulating the varied clinically relevant representations of UM for the evaluation of biology driven therapeutic approaches [13].

Patient-derived xenografts (PDXs) are cancer models established by engrafting and effectively propagating human tumor materials in animal hosts [14,15]. Currently, PDX models are generated using immuno-deficient mice and have become an important technique for preclinical assessment, medication guidance, and basic cancer research [16]. A PDX model grows in a 3D microenvironment, which includes vasculature that provides in vivo delivery of nutrients and oxygen, and host stromal cells which interact and communicate with the tumor cells. Compared to cell line-derived xenograft models, a PDX more closely recapitulates the heterogeneity of primary tumors and retains their gene-expression and mutation patterns [17,18,19,20]. Taken together, PDX is a promising preclinical model in personalized medicine, in which they may be used to predict patient-specific drug responses and guide patient therapies [21,22]. However, drug screens with mice PDX models are costly and time consuming.

In contrast, a zebrafish (*Danio rerio*) model requires much less material than a mouse model to assess drug efficacy, and allows for high-throughput screening, toxicity testing, convenient drug administration, and a short experimental duration [23,24]. Additionally, zebrafish possess numerous characteristics that make them an attractive model for human cancer research [25,26]. The adaptive immune system in zebrafish does not reach maturity until 4 weeks post-fertilization, allowing circumvention of graft rejection, without immune suppression [27]. Zebrafish have a comparable vertebrate anatomy and orthologues for 70% of human proteins, as well as paralogues of 84% of all known disease-related genes [28]. In addition, zebrafish embryos can absorb various small molecular weight compounds from water. Due to the extensive conservation of cancer-associated genes, zebrafish have emerged as promising organisms for modeling cancer in vivo. Currently, over 40 genetically engineered tumor models and many zebrafish xenograft models, including patient-derived xenografts in embryos and adult fish, have been established [29,30,31]. Therefore, the zebrafish PDX (zf-PDX) model can be considered a high-throughput intermediary between patients and mouse PDX models, and help to speed up the selection of promising anticancer drugs [32,33].

This technical study describes the development of a new and robust zf-PDX platform for UM research and efficient drug screening against UM. The proof-of-concept experiments provided a preclinical validation of drug toxicity and efficacy using this zf-PDX model. In future experiments, the model could be harnessed to facilitate the implementation of personalized medicine.

## 2. Results

### 2.1. Generation of Short-Lived Spheroid Cultures Derived from Primary UM Tissues

**Figure 1:**
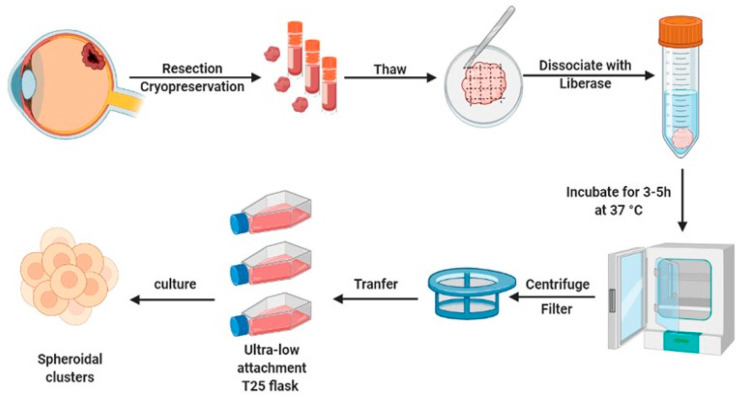
Schematic representation of patient–derived UM spheroid culture generation. The UM primary tissues were resected and frozen for storage or used directly. Tissue pieces were disaggregated, enzymatically dissociated, purified, and cultured in ultra-low adhesion plates, to form spheroids for further research.

UM is a rare disease, therefore biobanking of patient material is needed, to ensure optimal utilization of these precious tissue samples for both diagnostic and research purposes. To alleviate the scarcity of primary material (generally attained during enucleation), we have established a platform to isolate, preserve, and recover viable tissues. This was done by generating standardized protocols for cryopreservation and through the establishment of spheroid cultures derived from either fresh or cryo-preserved primary UM material (Figure 1). After surgical removal of the eye, UM tissue was resected from the surrounding tissues. The excised material was minced (fragments < 0.25 cm^3^) using scalpel blades and divided into cryogenic vials with neuronal stem cell (NSC) medium. The vials were put on ice, when used on the same day, or stored frozen at −80 °C in NSC medium, containing 10% DMSO, using a cell-freezing container (transferred to liquid nitrogen for long-term storage). The frozen tissues were thawed at 37 °C and mechanically disaggregated using a sterile scalpel blade with NSC medium, to prevent drying out. Subsequently, tissue pieces were enzymatically dissociated at 37 °C for 3–5 h with gentle agitation. The cell suspension was filtered and washed to remove extra-cellular matrix aggregates and plated in 24-well ultra-low adhesion plates (ULA). After 24 h, cellular aggregates were formed from fresh (*n* = 3) and frozen (*n* = 10) UM samples, with a 100% success rate. Although all samples were viable for extended periods, we observed little proliferation in these samples. Therefore, engrafted spheroid cultures were generally maintained only for the duration of the experimental procedure (<7 days). For biological replication, frozen bio-banked tissue from the same donor was used. Taken together, this protocol allows for the freezing and short-lived culturing of primary UM necessary for the experimental replications required for reliable drug screening with statistical power.

### 2.2. Short-Lived Patient-Derived UM Spheroids Maintain Their Melanocytic Origin

**Figure 2:**
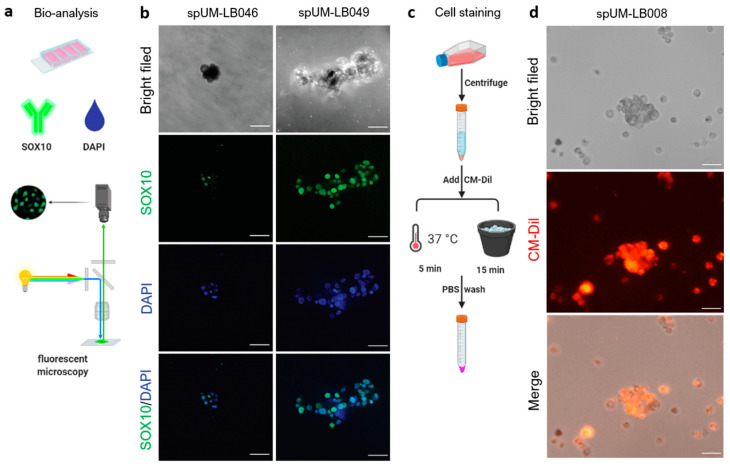
Analysis and transient chemical labelling of the patient-derived spheroids. (**a**) Workflow of immunostaining in the patient-derived spheroids. (**b**) Melanocyte marker SOX10 is expressed in spUM-LB046 and spUM-LB049. (**c**) Workflow of CellTracker staining of spheroids. (**d**) spUM-LB008 labeled using CM-Dil expressed red fluorescence and kept its spherical morphology for at least 5 days after staining. The images were taken with 20× magnification. The scale bar is 100 µm.

Next, we validated the biological properties of the patient-derived UM spheroids (Figure 2a,b). Our previous study indicated that the culturing of UM tissue as spheroids helped to preserve the expression of melanocyte-specific antigen (melan-A) and their intrinsic tumorigenic capacity [34]. Here, we determined the melanocytic origin of the cultured spheroids using the melanocyte marker SOX10 (Sry-related HMG-Box gene 10) (Figure 2b). SOX10 is a key nuclear transcription factor in the differentiation of neural crest progenitor cells into melanocytes [35]. SOX10 was used to stain patient-derived UM spheroids, to determine their melanocytic characteristics. As depicted in Figure 2b, positive SOX10 immunostaining identified that spUM-LB046 and spUM-LB049 spheroids maintained their melanocytic properties for at least 7 days in 3D culture. To stain single cells in spheroids, spUM-LB008 was labelled with CellTracker CM-Dil, a red fluorescent dye previously used to label cancer cells prior to engraftment into zebrafish [36,37]. Microscopic analysis indicated that CM-Dil (2 µM) is suitable for staining spUM-LB008 spheroids, with sufficient brightness for at least 5 days without inducing any morphological changes (Figure 2c,d). These findings indicated that chemically labeled cells derived from patient spheroids retained sufficient fluorescence for the duration of the experiments in the zebrafish xenograft model.

### 2.3. Spheroid-Derived Cells Successfully Engraft and Recapitulate Molecular Features of UM in Zebrafish Xenograft

**Figure 3:**
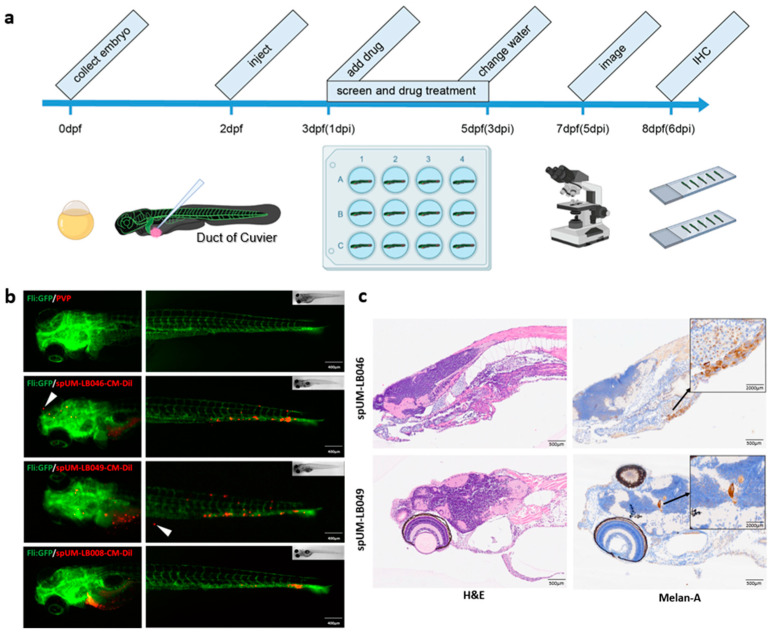
Establishment of zebrafish patient-derived xenograft model. (**a**) The timeline of drug treatment in the zebrafish xenograft assay: collection of embryos (0 dpf), UM cell injection (2 dpf), drug administration (1–5 dpi), imaging or IHC staining (6 dpi). (**b**) The fluorescent images of whole zebrafish engrafted without and with spheroid-derived cells at 5 dpi. The cells disseminated into the caudal hematopoietic tissue (CHT), while some cells remained at the injection site. The white arrows in the images of spUM-LB046 and spUM-LB049 point to extravascular cells. Images were taken with 20× magnification. The scale bar is 400 µm. (**c**) H&E and Melan-A staining of spUM-LB046 and spUM-LB049 at 6 dpi. The Melan-A images in black magnification showed the metastasis of UM cells in the brain of engrafted zebrafish embryos. The scale bar is 500 µm.

After the establishment, analysis, and fluorescent labeling of the patient-derived spheroids, we tested if the UM cells forming these spheroids retained their metastatic capacity after engraftment into zebrafish embryos. To address this issue, CM-Dil-labeled spheroids were dissociated into a single cell suspension by repetitive pipetting, and red fluorescent CM-Dil stained UM cells were intravenously injected into the duct of Cuvier (doC) of transgenic *(fli:GFP) Casper* zebrafish larvae with green vasculature at 2 days post-fertilization (dpf). Use of CM-Dil may, however, lead to the formation of artefacts, and this was controlled for in data analysis through size gating (objects < 20 um were excluded). The presence of UM cells was confirmed at 6 dpf with anti-melan-A immunostaining. Subsequently, the head and tail regions were imaged at 6 days post injection (dpi) (Figure 3a). Upon engraftment, UM cells obtained from spUM-LB046, spUM-LB049, and spUM-LB008 spheroids disseminated hematogenously; after 6 days, metastatic foci were observed in the caudal hematopoietic tissue (CHT). In some embryos, cells could also be found in the head and at the injection site (Figure 3b). In the embryos engrafted with spUM-LB046- and spUM-LB049-derived cells, a few extravascular cells were detected. These results indicated that the spUM-LB046 and spUM-LB049 cells derived from the primary patient spheroids maintained their metastatic potential. To further validate the UM malignancy in the xenografts, we performed IHC staining of embryos engrafted with spUM-LB046 and spUM-LB049. The expression of Melan-A could be detected in the heads of zebrafish from both injected groups (Figure 3c), implying that the spUM-LB046- and spUM-LB049-derived cells retained melanocytic properties in the xenografted embryos. Moreover, these results demonstrated that, at 5 dpi, the engrafted cells were still present within the engrafted zebrafish and that these cells maintained the same markers, both in vitro and in vivo, justifying the usefulness of this model for drug screening and preclinical assessment of an anti-tumor response.

### 2.4. Zebrafish Model Allows Versatile Drug Toxicity Testing with Phenotypic Profiling

**Figure 4:**
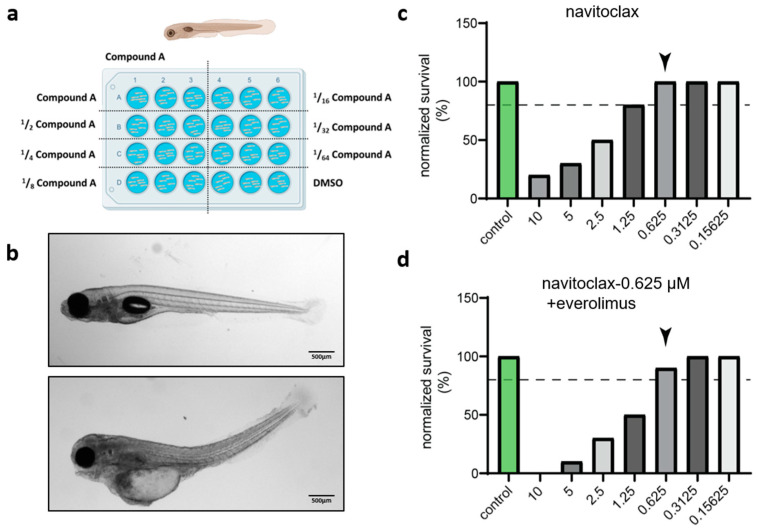
The maximum tolerated dose (MTD) of drug in the wild-type zebrafish. (**a**) Schematic diagram of the experimental set up for drug toxicity in wild-type zebrafish embryos: drug administration (3–7 dpf) and measurement of MTD (8 dpf). (**b**) Representative images of normal (top) and malformed (bottom) zebrafish larvae at 8 dpf. (**c**) The dotted line denotes the 80% survival rate used as a cut-off for the establishment of the MTD. The survival of zebrafish treated with navitoclax top exceeded 80% at concentration below 0.625 µM. The black arrow indicates the MTD of navitoclax (0.625 µM). (**d**) The black arrow indicates the MTD of navitoclax (0.625 µM) + everolimus (0.625 µM) when treated through bath submersion administration.

In order to test the effectiveness of antineoplastic drugs in the zf-PDX model, we first determined their toxicity on wild-type, non-injected embryos. We selected drugs with known efficacy against UM cells and the murine subcutaneous UM PDX model [38,39]. We selected navitoclax, a small molecule inhibitor, which targets members of the B-cell chronic lymphocytic lymphoma 2 (BCL-2) family of apoptotic receptors, including BCL-XL [40], and everolimus, a small molecule drug that inhibits mammalian target of rapamycin (mTOR) [41]. Considering that small molecules can be actively absorbed from water by the embryos, we chose water administration (WA) as a convenient and rapid way to explore drug toxicity through phenotypic profiling. As described in Figure 4a, at 3 dpf, the embryos (6 individuals per well, 3 wells per condition) were exposed to different concentrations of navitoclax (≤10 µM, 2-fold serial dilution). The control group was treated with the same concentration of solvent (dimethylsulfoxide, DMSO) as used in the navitoclax group. The water with added drugs was changed every other day until 7 dpf, and the wellbeing of embryos was monitored daily using microscopic examination. Figure 4b shows a representative image of both a normal and malformed zebrafish larva, the latter clearly distinguishable through its bent tail and profound edema. The maximum tolerated dose (MTD) was determined as the drug concentration at which at least 80% of treated individuals survived without an aberrant phenotype. The MTD of navitoclax was 0.625 µM (Figure 4c). In order to establish the MTD for combined treatment, we repeated the toxicity assay with different concentrations of everolimus (≤10 µM, 2-fold serial dilution) on top of the MTD of navitoclax (Figure 4d). Zebrafish embryos tolerated the combination of 0.625 µM navitoclax and 0.625 µM everolimus, with only minor side effects.

### 2.5. Combination Treatment with Navitoclax and Everolimus Validates UM zf-PDX Model as a Versatile Preclinical Tool for Anti-UM Drug Sceening

Clinical trials relying on mono-therapeutic strategies did report any significant benefit in terms of the overall survival of UM patients [4,42,43]. Extensive screens of 30 dual drug combinations in a panel of eight UM cell lines found that while BCL-2/BCL-xl inhibitor navitoclax (ABT263) as a single-agent exhibited low efficacy in UM cell lines, this drug sensitized the tested cell lines to mTOR, MEK, and MDM2 inhibitors, including mTORC1 inhibitor-everolimus (RAD001) [38]. To validate the feasibility of the UM zf-PDX model as a new drug screening platform, we therefore selected navitoclax and everolimus for mono and combination treatments in a proof-of-concept experiment.

**Figure 5:**
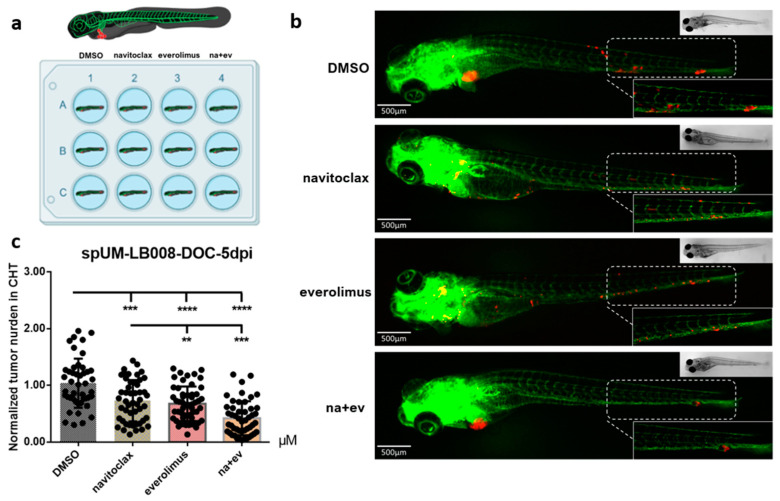
Navitoclax and everolimus and the combination of both chemotherapeutic agents reduced spUM-LB008 tumor burden in zf-PDX model. (**a**) The experimental layout of drug treatment in the UM zf-PDX model. (**b**) Representative phenotypes of zebrafish in the DMSO-control and drug treatment groups at 6 dpi (*n* = 3, *p* < 0.01). *P* values were indicated as follows: ** *p* < 0.01, *** *p* < 0.001, **** *p* < 0.0001. (**c**) Effect of navitoclax, everolimus, and their combination treatment on tumor burden, compared to the control group. The combination of navitoclax and everolimus reduced the tumor burden significantly compared to everolimus and navitoclax alone. The fluorescence intensity in CHT (white rectangle) of each individual zebrafish was measured as the metastasized tumor burden. The scale bar is 500 µm.

Figure 5a shows the experimental layout of drug treatment in the zf-PDX model. Tumors spheroids were injected into the duct of Cuvier at 2 dpf and positive screened individuals at 3 dpf. Embryos engrafted with CM-Dil-labelled cells acquired from spUM-LB008 spheroids were treated (6 embryos per well in triplicate) with DMSO, navitoclax, everolimus, and a combination of navitoclax and everolimus (18 embryos per group), all at their MTD. Zebrafish in both the control and drug-treated groups showed no physical malformations and displayed normal swimming behavior and lateral line responses. Representative phenotypes of the different treatments at 6 dpi are depicted in Figure 5b. At the experimental endpoint (6 dpi), the CM-Dil-fluorescence intensity in CHT of each individual zebrafish was measured using stereo fluorescence microscopy. The fluorescence values considered as relative tumor burden were normalized to the control DMSO group (Figure 5c). Single treatments with navitoclax and everolimus reduced the tumor burden to 71% and 64%, respectively. Combinatorial treatment showed a reduction effect (62%) that was significantly different compared to the single treatments. The drug efficacy obtained in this proof-of-concept experiment in the zf-PDX model recapitulated published results using UM cells and the murine PDX model [38]. Our recent study harnessing this model identified ferroptosis as a new and druggable pathway for the treatment of UM patients [34]. In another group, Glinkina et al. also tested target agent combinations with the zf-PDX UM model but did not detect tumor regression [44]. Overall, our results validated the feasibility and robustness of the versatile UM zf-PDX model for preclinical drug screening and preclinical evaluation of personalized therapy response.

## 3. Discussion

UM is a rare and deadly cancer, and due to its low incidence, tissue samples from UM patients are scarce and of irreplaceable value for research. Therefore, we developed a method to make optimal use of the limited number and size of the available samples. In this study, we have generated a platform to isolate, preserve, and transiently recover viable patient-derived UM tissues through the generation of short-lived spheroid cultures. These cultures retain UM properties (for at least 7 days), allowing engraftment into zebrafish embryos to test anti-UM drugs by measuring the inhibition of metastatic dissemination and colonization. Importantly, the option of sequential re-establishment of spheroids from frozen tissue acquired from the same patient enables the experimental replications mandatory for reliable drug screening with statistical power. The zebrafish, as a preclinical screening model, is perfectly suited for this purpose [45].

The established zf-PDX model derived from short-lived UM cultures offers several key advantages compared to time-consuming and expensive murine models. The short generation time, the large number of offspring, and the small size of the embryos make zebrafish larvae a more practical and less expensive animal model. These features enable high-throughput drug screening, using a limited tumor volume [46,47,48,49]. Furthermore, the transparency of the embryos facilitates the visualization of tumor cell behavior and its interactions with the microenvironment, such as host blood vessels, immune cells, and stromal cells [50]. Considering that most UM metastasize hematogenously, zebrafish provide a visible tractable model of UM invasion, extravasation, and angiogenesis [51,52].

Clinical data from the LUMC indicate that spUM-LB046 and spUM-LB049 are BAP1 (BRCA1 associated protein 1) mutant. BAP1 is a tumor suppressor gene and its encoded enzyme binds to the breast cancer type 1 susceptibility protein (BRCA1) via the RING finger domain of the latter and binds to BRCA1 and BARD1, forming a tumor suppressor complex mapped to chromosome 3 (3p21.31–p21.2) region, which is generally deleted in metastatic uveal melanoma [53]. In line with this, metastases were detected in the patients themselves, whereas spUM-LB008 is BAP1-positive and its donor did not present with UM metastasis (the details can be seen in the Supplementary Table S1). These data underscore the predictive capacity of the zebrafish UM model and are consistent with the clinical predictive marker (BAP1 loss). Furthermore, this model not only allows the exploration of the underlying molecular mechanisms of UM metastasis, but also the preclinical assessment of drug sensitivities for individual patients. Therefore, we reason that this model provides a path towards the personalized treatment of patients with rare tumors [54,55,56].

Recent technological advances in the generation of transplantation-based zebrafish cancer avatars combined with their intrinsic logistic advantages (scale, cost, time, and multiplexing of conditions) move zebrafish to the forefront of phenotype-based testing of drug responses for precision medicine [30,37,57]. Especially in clinical trials, the use of zebrafish has led to several valuable preclinical discoveries. For instance, in 2019, the first larval zf-PDX co-clinical trial was initiated, and olaparib plus temozolomide treatment was tested in an adult zf-PDX xenograft model of rhabdomyosarcoma. This therapy was transferred to a clinical trial without additional prerequisite models [48]. As for cutaneous melanoma, a present, an on-going phase II clinical trial of leflunomide combined with vemurafenib is the first to stem from an initial screen in zebrafish [58]. Many small molecules observed to have disease-rescuing activity in zebrafish have made it into clinical trials [59].

Additionally, the zebrafish has been shown to hold much promise for providing new insights into micro-environmental interactions between host and cancer cells [60,61,62]. However, despite these many advantages over other animal models, some challenges remain when working with the zf-PDX model. One challenge is the difference in drug administration. Zebrafish larvae are usually treated by adding drugs directly to the water. Drug delivery in larvae via water exposure makes it difficult to accurately assess drug dosing, pharmacokinetics, and pharmacodynamics [63]. Pharmacokinetic differences between humans, mice, and zebrafish are important concerns for the field. Facing this problem, some researchers have used dose-conversion factors for submersion therapy [64]. Another solution is to administrate drugs through intravenous injection. This is more similar to drug treatment in humans, but is challenging because of the size of zebrafish [65].

There is a clear consensus that no single preclinical model can substitute for actual human trials. Therefore, are taking advantage of the inherent strengths of the zebrafish PDX model to improve their translational relevance, for the ultimate benefit of the UM patients.

## 4. Materials and Methods

### 4.1. Tissue Collection and Cryopreservation

Patients’ samples spUM-LB046, spUM-LB049, and spUM-LB008 were provided by M.J. Jager, Leiden University Medical Center (LUMC). Fresh material was used for spheroids under the METC protocol UM CURE 2020: Prospective collection: new treatment options for metastatic uveal melanoma (NL57166.058.16). All patients signed an informed consent form. Tumor material obtained after enucleation of the eye was placed in a Petri dish containing 5 mL Complete NeuroCult Basal Medium (NC) (the composition of the complete medium is shown in Supplementary Table S2). The tumor material was minced with sterile scalpel blades and the minced tissue was divided into prepared cryogenic vials with NC medium, containing 5 mg/mL Primocin (Invivogen, San Diego, CA, USA). Care was taken to ensure that all material was submerged. The vials were put on ice when used on the same day. When tissue was to be processed at a later timepoint, tumor tissues was frozen, after the addition of 10% (*v*/*v*) dimethyl sulfoxide (DMSO, Sigma-Aldrich, Burlington, VT, USA) to the NC medium. The cryovials containing the tissues were placed in an isopropanol chamber and stored at −170 °C.

### 4.2. Patient-Derived Spheroids Disaggregation and Culture

The frozen patient-derived tissues were thawed at 37 °C and transferred to basal NeuroCult medium (bNC) containing 5 mg/mL Primocin. The tissue mass was collected in a 50 mL centrifuge tube, the volume was adjusted to 10 mL with bNC and supplemented with 0.01 mg/mL Liberase TL (Roche, Basel, Switzerland). The suspended tumor tissues were incubated in a shaker (Salm and Kipp, Utrecht, The Netherlands) at 37 °C for 3–5 h, while shaking vigorously (250 rpm). The tubes were vortexed intermittently during this incubation, to disperse any tissue aggregates. The disaggregation process was checked, until there were no visible tissue clusters remaining. The cell suspension was filtered through a sterile 30 µm cell strainer, to remove all cell and extra-cellular matrix aggregates. Cells were pelleted and suspended in complete NC medium. The suspension was diluted and plated in an ultra-low attachment T25 flask (Corning, Wiesbaden, Germany). After several days of culture, the cells generally coalesced into larger cell aggregates (spheroids) and were disrupted prior to labeling and engraftment.

### 4.3. CellTracker Labelling

The patient-derived spheroid suspension was centrifuged (1000× *g*, 5 min), and the supernatant was removed. Then cells were resuspended in 3 mL TrypLE (Thermo Fisher Scientific, Waltham, MA, USA) for 10 min at 37 °C, combined with intermittent agitation with a 1000 µL pipette, and physically dissociating cell aggregates by pipetting up and down. TrypLE was inactivated with the addition of 7 mL complete NC medium, followed by centrifuging and removing the supernatant. The spheroids were resuspended with phosphate buffered saline (PBS, Sigma-Aldrich, Burlington, VT, USA) containing 2.5 µM CellTracker CM-Dil dye (Thermo Fisher Scientific [C700], Waltham, MA, USA) in a 15 mL tube. Cells were incubated in the dark for 5 min at 37 °C and then for an additional 15 min at 4 °C in a dark environment. After labeling, the CM-Dil reagent was removed through centrifugation. The cells were washed with PBS and resuspended in fresh complete NC medium.

### 4.4. Immunofluorescence Staining

The spheroids were seeded using a cut back 1000µL pipette tip into a multi-chambered slide in Vitrogel (The Well Bioscience, North Brunscwick, NJ, USA) diluted 3-fold in culture medium, immediately prior to seeding. The Vitrogel matrix was left to polymerize for at least 30 min at 37 °C. Spheroids were fixed overnight through incubation with 4% paraformaldehyde (PFA, Sigma-Aldrich, Burlington, VT, USA) at 4 °C. After fixation, the PFA was removed and the spheroids embedded in Vitrogel were washed thoroughly with PBS containing 200 mM glycine at room temperature. For subsequent immunostaining the liquid from each chamber was carefully aspirated, so as not to disturb the spheroids embedded in the gel. Samples were permeabilized for 30 min at room temperature with 0.3% Triton-X100 (diluted in DPBS without calcium and magnesium). The chambers were washed thoroughly with PBS containing 0.05% Tween (Sigma-Aldrich, Burlington, VT, USA) (PBS-T). The samples were blocked with PBS-T containing 5% goat serum and were incubated for 60 min at room temperature. The solution from the wells was carefully removed and the cells were incubated with rabbit anti-human SOX10 primary antibody (Abcam [SP267], Cambridge, UK), diluted in PBS-T containing 5% goat serum solution at 2–8 °C overnight. After extensive washing, the cells were then treated with a secondary antibody, goat-anti rabbit Alexa Fluor 488 diluted in PBS-T containing 5% goat serum solution, for 60 min at room temperature. The cells were washed with PBS-T with calcium and magnesium three times and stained with a 4′,6-diamidino-2-phenylindole (DAPI) solution (3 ng/mL) for 10 min [66,67]. The slide was imaged under an inverted Leica TCS SPE confocal microscope (Leica Microsystems, Wetzlar, Germany).

### 4.5. Zebrafish Embryo Preparation

Transparent Tg *(fli:GFP) Casper* zebrafish were housed under standard conditions at 28.5 °C. The zebrafish breeding pairs were set up with an equal ratio of females and males in breeding tanks for mating overnight. The embryos were collected the next morning and maintained in a 28.5 °C incubator. Unfertilized embryos and malformed larvae were removed at 1 and 2 dpf.

### 4.6. The Maximum Tolerated Dose (MTD) Assay

To determine the MTD of navitoclax and everolimus administered in water, a 2-fold dilution series was prepared (10 µM to 156 nM) and compared to the highest volume of DMSO added as a negative control. At 3 dpf, corresponding to 1 dpi in injected larvae, the larvae were randomly divided into 6 groups and put in 24-well plates with egg water containing either drugs or DMSO (6 individuals per well; 1 mL zebrafish water per well). The compounds were diluted in zebrafish water prior to administration. The embryos were maintained at 34 °C and monitored daily. The egg water with drugs was changed every other day. The dead embryos were carefully removed when changing the zebrafish water containing the drugs, and survival was recorded at 5 dpi.

### 4.7. Duct of Cuvier Injection

Needles for zebrafish doC injection were prepared prior to engraftment; needles were manually shortened to an approximate opening of 20 µm. The labeled cells were gently but thoroughly resuspended in a 15 mL tube to ensure the homogeneity of the cell-mixture prior to the loading of every needle. The cell suspension was backloaded into the needle using a micro-loader pipette tip (Eppendorf, Hamburg, Germany). The needle was inserted into a micro manipulator (World Precision Instruments, Sarasota, FL, USA).

Embryos were incubated in a 34 °C incubator for 2 h before injection, to facilitate shedding of the chorion. The dechorionated embryos were strained out and anesthetized with 0.002% tricaine. Anesthesia time was kept under 2 h, to ensure survival of the embryos. The anesthetized embryos were transferred onto an agarose dish (1.5% agarose in zebrafish water) and all superfluous water was removed. The embryos were injected via the posterior point of the doC from the dorsal side, where the duct of Cuvier forms a large collecting blood vessel leading up to the heart. The ejection pressure and pneumatic pulse time were adjusted to ensure about 300–500 cells were injected per larva. The injected larvae were flushed off with fresh zebrafish water and transferred to a new clean Petri dish. Care was taken during the injections to ensure that the larvae did not dry out. The injected embryos were selected 16 h post injection, using a fluorescent microscope according to phenotypic normalcy and the presence of red signal in the circulation.

### 4.8. Immunohistochemistry Analysis of Engrafted Zebrafish Larvae

Prior to immunohistochemical analysis, the engrafted zebrafish larvae were euthanized with tricaine and fixed for 16 h in ice cold 4% paraformaldehyde in PBS. After fixation, larvae were washed with PBS containing 0.05% tween 20 (*v*/*v*) and 200 mM Glycine. Larvae were stored in the dark at 4 °C, until further processing. Fixed zebrafish larvae were arrayed in a grid and embedded in agarose. Care was taken to ensure equal localization in the *x*, *y*, and *z* axes. Larvae were sectioned along the ventral axis. Sections were cut at 4 µm from formalin-fixed paraffin embedded blocks of UM cell-containing zebrafish, as detailed above, and placed onto X-tra adhesive slides (Leica Biosystems, Milton Keynes, UK). Immunohistochemical (IHC) staining was performed using the Bond RXm Automated Stainer with high pH antigen retrieval and Bond polymer-refine detection systems in either red or brown chromogen [68]. Primary antibodies included mouse anti-melan-A (Agilent Technologies, Santa Clara, CA, USA) at a concentration of 1 µg/mL. Slides were counterstained with hematoxylin and mounted with a resin-based mountant. Human UM tissue was used as a positive control for each of the primary antibodies. Mouse IgG1 isotype control at a concentration of 1 µg/mL was also included in each assay [69].

### 4.9. Drug Treatment through Water Administration

For drug treatment, implanted larvae were randomly divided into 4 groups and transferred to 12-well plates with egg water containing drugs or DMSO control (6 individuals per well; 1 mL egg water per well). At 1 dpi, candidate drugs were dissolved into egg water for further administration. The embryos were maintained at 34 °C and monitored daily. Dead embryos were removed, to ensure that they did not negatively influence the survival of the other larvae. Drug-containing egg water was changed every other day.

### 4.10. Whole Body Zebrafish Larval Imaging

Larvae were anaesthetized and transferred to a 1.5% agarose dish at 5 dpi. The zebrafish water was removed after the larvae were imaged using a stereo fluorescence microscope. The zebrafish larvae were oriented in the middle of the image, gills facing downwards. The microscope settings were adjusted (i.e., fluorescent exposure, gain, intensity) to be almost saturated, in the control group. The settings were kept constant during imaging. After imaging, the larvae were fixed for the subsequent IHC analysis.

### 4.11. Statistical Analysis

All images were analyzed in ImageJ/Fiji [70]; measuring the CHT fluorescence density of each individual image under identical threshold conditions as set on the control population. Statistical analysis was performed using GraphPad (Prism 6). Outliers were tested for in the collect raw data (Q5) and whenever present they were removed from all populations. All measurements are either normalized to day 1 or to the control (DMSO) treated groups. A one-way analysis of variance (ANOVA) was used for statistical analysis. Graphs display the mean ±SD. Differences were considered significant when *p* < 0.05 (* *p* < 0.05, ** *p* < 0.01, *** *p* < 0.001, **** *p* < 0.0001).

## 5. Conclusions

**Figure 6:**
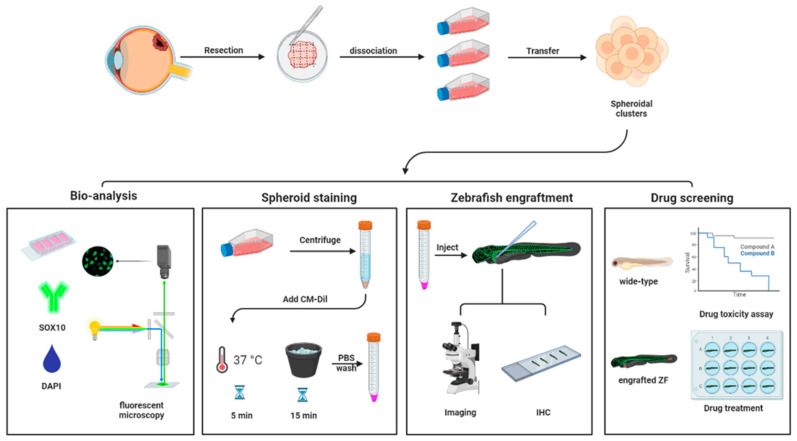
zf-PDX model derived from short-lived UM spheroids for drug discovery. After generation of patient-derived spheroids, ex vivo analysis was used to detect their tumorigenic and melanocytic characteristics. Transient cell staining was used to visualize the cell distribution in the zebrafish UM-PDX model. The drug screening using zf-PDX enabled evaluation of anti-UM drugs in a preclinical setting (Created with BioRender.com).

This technical study demonstrated the zf-PDX model as a robust and reliable platform for UM research and the screening of potential anti-UM drugs (Figure 6). Here, we have provided a step-by-step methodological description of this pipeline. First, we confirmed that the generation of short-lived spheroid cultures derived from primary UM tissues is feasible, as previously published by our group [71]. The SOX10 immunostaining validated that the spheroids maintained their composition of melanocytes over time. After disaggregation and transient staining, the spheroid suspension was injected into the circulatory system of zebrafish, and we demonstrated that we could trace the formation of metastatic foci in the tail of the engrafted larvae. The expression of Melan-A in larvae proved that the engrafted UM cells could survive, metastasize, and still possessed melanocytic characteristics in zebrafish. The applicability of this zf-PDX model for anti-UM drug screening was demonstrated in the proof-of concept study, using the BCL-2/BCL-xl inhibitor, navito-clax, and the mTORC1 inhibitor, everolimus, which were effective in murine-PDX models [38]. The combination of navitoclax and everolimus significantly reduced the tumor burden in the zebrafish, validating that our zf-PDX model is relevant and a versatile tool for drug screening. 

Overall, this zf-PDX model not only enables the exploration of the underlying molecular mechanisms of UM metastasis, but also provides great promise for the rapid preclinical assessment of drug sensitivities for individual patients. We propose that this model provides a path towards the personalized treatment of patients with UM and other rare tumors.

## Data Availability

Data are contained within the article.

## Supplementary Materials

The following supporting information can be downloaded at: https://www.mdpi.com/article/10.3390/ph16040598/s1, Table S1: Characteristics of uveal melanoma used in zf-PDX models; Table S2: The composition of Complete NeuroCult Medium.
