# Supplementary material for: Zebrafish Patient-Derived Xenograft Model as a Preclinical Platform for Uveal Melanoma Drug Discovery
Source: Pharmaceuticals (Basel). 2023 Apr 15;16(4):598. doi: 10.3390/ph16040598 (PMC10141637; doi:10.3390/ph16040598)
Supplement: Supplementary file 1 [file pharmaceuticals-16-00598-s001.zip › Supplementary table S1.pdf]

Supplementary table S1. Characteristics of uveal melanoma used in zf-PDX models

	**Male/ Female**	**Age**	**Eye color**	**Diagnosis**	**Cell type**	**BAP1 protein expression**	**Chromosome 3**	**Chromosome 8q copies**	**Metastasis Yes/No**	**Follow-up months**	**Pigment**
spUM-LB046	Male	59		Choroid	Epi	Loss	M3	4	Yes	47†	Heavy
spUM-LB049	Male	42	Blue	Choroid	Epi	Loss	M3	3-4	Yes	46†	None
spUM-LB008	Male	75	Blue	Choroid	Mix	No loss	M3	2	No	25	Moderate

Epi = Epithelioid cell type, Mix = mixed spindle and epithelioid cell type

M3: monosomy of chromosome 3
