# Supplementary material for: Zebrafish Patient-Derived Xenograft Model as a Preclinical Platform for Uveal Melanoma Drug Discovery
Source: Pharmaceuticals (Basel). 2023 Apr 15;16(4):598. doi: 10.3390/ph16040598 (PMC10141637; doi:10.3390/ph16040598)
Supplement: Supplementary file 1 [file pharmaceuticals-16-00598-s001.zip › Supplementary table S2.pdf]

Supplementary table S2. The composition of Complete NeuroCult Medium

Complete NeuroCult Medium-500 mL

Component	volume
NeuroCult NS-A Basal Medium (StemCell CAT# 05750)	480 mL
B27 (Gibco 12587-010)	10 mL
N2 supplement (Gibco 17502-048)	5 mL
100x Glutamax (Gibco/Life Technologies 35050-038)	5 mL
Primocin (penicillin/streptomycin)	1 mL
EGF (1 mg/mL stock, final 20 ng/ml; Peprotech AF-100-15)	10 µL
bFGF (1 mg/mL stock, final 20 ng/ml; Peprotech 100-18B)	10 µL
heparin (5000 IE/ml from Pharmacy VUMC)	500 µL
